# Evaluation of Pathway Activation for a Single Sample Toward Inflammatory Bowel Disease Classification

**DOI:** 10.3389/fgene.2019.01401

**Published:** 2020-02-05

**Authors:** Xingyi Li, Min Li, Ruiqing Zheng, Xiang Chen, Ju Xiang, Fang-Xiang Wu, Jianxin Wang

**Affiliations:** ^1^School of Computer Science and Engineering, Central South University, Changsha, China; ^2^Neuroscience Research Center & Department of Basic Medical Sciences, Changsha Medical University, Changsha, China; ^3^Department of Mechanical Engineering and Division of Biomedical Engineering, University of Saskatchewan, Saskatoon, SK, Canada

**Keywords:** similar complex diseases, pathway activation, single sample, inflammatory bowel disease, pathway biomarkers

## Abstract

Since similar complex diseases are much alike in clinical symptoms, patients are easily misdiagnosed and mistreated. It is crucial to accurately predict the disease status and identify markers with high sensitivity and specificity for classifying similar complex diseases. Many approaches incorporating network information have been put forward to predict outcomes, but they are not robust because of their low reproducibility. Several pathway-based methods are robust and functionally interpretable. However, few methods characterize the disease-specific states of single samples from the perspective of pathways. In this study, we propose a novel framework, Pathway Activation for Single Sample (PASS), which utilizes the pathway information in a single sample way to better recognize the differences between two similar complex diseases. PASS can mainly be divided into two parts: for each pathway, the extent of perturbation of edges and the statistic difference of genes caused by a single disease sample are quantified; then, a novel method, named as an AUCpath, is applied to evaluate the pathway activation for single samples from the perspective of genes and their interactions. We have applied PASS to two main types of inflammatory bowel disease (IBD) and widely verified the characteristics of PASS. For a new patient, PASS features can be used as the indicators or potential pathway biomarkers to precisely diagnose complex diseases, discover significant features with interpretability and explore changes in the biological mechanisms of diseases.

## Introduction

Complex diseases threaten human health and life quality. Similar complex diseases make the early diagnosis of patients more difficult due to similar clinical symptoms. Therefore, mining effective biological information to accurately discriminate between similar complex diseases has become the most important research area of biomedicine. In the previous research, several methods based on a single biological network, such as the metabolic network, regulatory network, or protein–protein interaction (PPI) network, have been put forward to aid in disease prediction, diagnosis, prognosis, and so on (Winter et al., 2012; Cun and Fröhlich, 2013). Nevertheless, these methods are not robust because of the low reproducibility (Yousefi and Dougherty, 2012; Amar et al., 2015; Choi et al., 2017) that results from the cellular heterogeneity within tissues, the heterogeneity of samples, and errors of measuring technologies.

Since genes generally take effect synergistically by forming functional modules, inferring features related to disease classification at the functional level can effectively ameliorate the adverse effects of heterogeneity and obtain more reproducible markers. Some methods utilize Gene Ontology (Ashburner et al., 2000) to differentiate disease states (Zhang et al., 2017) while others integrate pathway information. Pathways reflect biological processes within cells, such as metabolism, signaling, and growth cycles, and markers identified based on pathway information can thus maintain functional interpretability (Haider et al., 2018). Moreover, the occurrence and progression of complex diseases, such as inflammatory bowel disease (IBD), are often related to the dysregulation of significant pathways. Discovering the involved pathways and quantifying their disorders are of great significance in understanding complex diseases (Bild et al., 2006; Thomas et al., 2008; Markert et al., 2011; Drier et al., 2013).

A series of methods for disease classification integrate pathway information from the Molecular Signatures Database (MSigDB) (Subramanian et al., 2005) or Kyoto Encyclopedia of Genes and Genomes (KEGG) (Kanehisa and Goto, 2000). Several works extract significant features from the genes along pathways to distinguish diseases (Huang et al., 2003; Bild et al., 2006; Lee et al., 2008a; Young and Craft, 2016). Although these works can combine pathway information to classify diseases effectively, they only regard a pathway as a set of genes and ignore the edge information between genes, which may lead to the loss of important information related to diseases. To overcome this problem, some methods for analyzing the intrinsic structures of pathways and integrating topological characteristics of pathways have been proposed (Liu et al., 2013; Han et al., 2017). These existing algorithms can effectively utilize the topological information of pathways to predict disease status. Nevertheless, none of them assesses condition-specific states for each patient from a pathway perspective, but this is essential to revealing the molecular mechanisms of complex diseases at the system level.

By analyzing the high-dimensional information of expression data and the differential distribution (i.e., volcano distribution) of a single patient against a given number of normal samples (Liu et al., 2016), we propose a novel framework to classify two similar complex diseases by evaluating the pathway activation based on single sample analysis. Our method consists of two steps: (1) a fully connected network for each pathway is constructed and the perturbation of each edge in the network caused by the introduction of each disease sample is evaluated. For all genes in the pathways, the statistical difference of gene expression between a single disease sample and normal samples is evaluated; (2) a novel method named as AUCpath is introduced to evaluate the pathway activation for single sample (PASS) of each pathway from both node and edge aspects, which converts the high-dimensional, small-sample gene expression matrix into a PASS matrix. Finally, a random forest classifier based on PASS features is built to examine the classification performance.

We applied PASS to classify ulcerative colitis (UC) and Crohn's disease (CD) (Ananthakrishnan, 2015). UC and CD have many common clinical features, such as abdominal pain, diarrhea, recurrent episodes, and so on. They are therefore collectively referred to as IBD. IBD is a special kind of intestinal inflammatory disease caused by common factors such as genetics, environmental triggers, immunoregulatory defects, and microbial exposure (Hanauer, 2006). Currently, there is no gold standard for discriminating UC and CD, but the responses and effects after medication of these two complex diseases are not the same (Akobeng et al., 2016; Baumgart and Sandborn, 2007), and this has motivated many attempts to understand the differences in the molecular characteristics between these two similar complex diseases at the tissue level (Lawrance et al., 2001; Burczynski et al., 2006; Wu et al., 2007). The improved understanding of the differential mechanisms of UC and CD from a molecular perspective can improve the diagnostic accuracy and have the potential to improve the therapeutic effect and the success rate of clinical trials.

We compare our method with seven network-based, GO-based, and pathway-based methods, respectively, and obtain prominent performance against these methods. In addition, our experimental results showed that our method can elucidate the molecular mechanism of UC and CD and has the potential to identify biomarkers with functional interpretability.

## Materials and Methods

### Dataset and Preprocessing

We downloaded two pediatric datasets and three adult datasets from the Gene Expression Omnibus (GEO) (Edgar et al., 2002), namely GSE9686 (Carey et al., 2007), GSE3365 (Burczynski et al., 2006), GSE36807 (Montero-Meléndez et al., 2013), GSE71730 (Gurram et al., 2016), and GSE16879 (Arijs et al., 2009). All of them contain UC, DC, and normal samples.

In order to maintain the consistency of data and reduce the impact of noise, we selected data from the same anatomical site and patients under the same conditions. We excluded samples of CD patients during treatment for GSE9686 and samples of Crohn's ileitis for GSE16879. We mapped probes to gene ID using files provided by the corresponding platforms, discarded probes corresponding to multiple genes, and chose the median when multiple probes were mapped to the same gene to eliminate the influence of measurement errors. Only genes detected in all datasets can be used for the downstream analysis. As a result, there were 11242 genes included in all five datasets. **Table 1** summarizes the above datasets.

**Table 1:** Summary of the gene expression datasets.

Name	Healthy	UC	CD	Total genes	Type of samples	Reference	URL
GSE9686	8	5	11	15747	Pediatric samples	(Carey et al., 2007)	https://www.ncbi.nlm.nih.gov/geo/query/acc.cgi?acc=GSE9686
GSE3365	42	26	59	12432	Adult samples	(Burczynski et al., 2006)	https://www.ncbi.nlm.nih.gov/geo/query/acc.cgi?acc=GSE3365
GSE36807	7	15	13	20486	Adult samples	(Montero-Meléndez et al., 2013)	https://www.ncbi.nlm.nih.gov/geo/query/acc.cgi?acc=GSE36807
GSE71730	10	15	22	20486	Pediatric samples	(Gurram et al., 2016)	https://www.ncbi.nlm.nih.gov/geo/query/acc.cgi?acc=GSE71730
GSE16879	6	24	19	20486	Adult samples	(Arijs et al., 2009)	https://www.ncbi.nlm.nih.gov/geo/query/acc.cgi?acc=GSE16879

From the KEGG database, all human pathways were downloaded using the KEGGgraph package (Zhang and Wiemann, 2009). A total of 294 pathways were extracted. Each pathway consisted of a set of genes and their interactions; genes were represented by nodes, and interactions were edges in the KEGG human pathways. Genes that were not present in the expression profiles and their corresponding interactions were discarded. Considering the following analysis, pathways containing only one edge were not included. Finally, 291 pathways were retained, and these contained 3926 genes in total.

### Pathway Activation for Single Sample

Pathway-based features are more robust while maintaining biological interpretability and tend to be small in number, which can prevent overfitting. In this study, we introduced a new method, called PASS, to evaluate the state of each known pathway. PASS defined the state of a pathway from the aspect of genes and regulatory links. Although it was difficult to analyze the regulatory links in the pathway for each patient, the sample-specific network (SSN) analysis provided a feasible and effective way to mine the different regulatory patterns for each patient.

**Figure 1:**
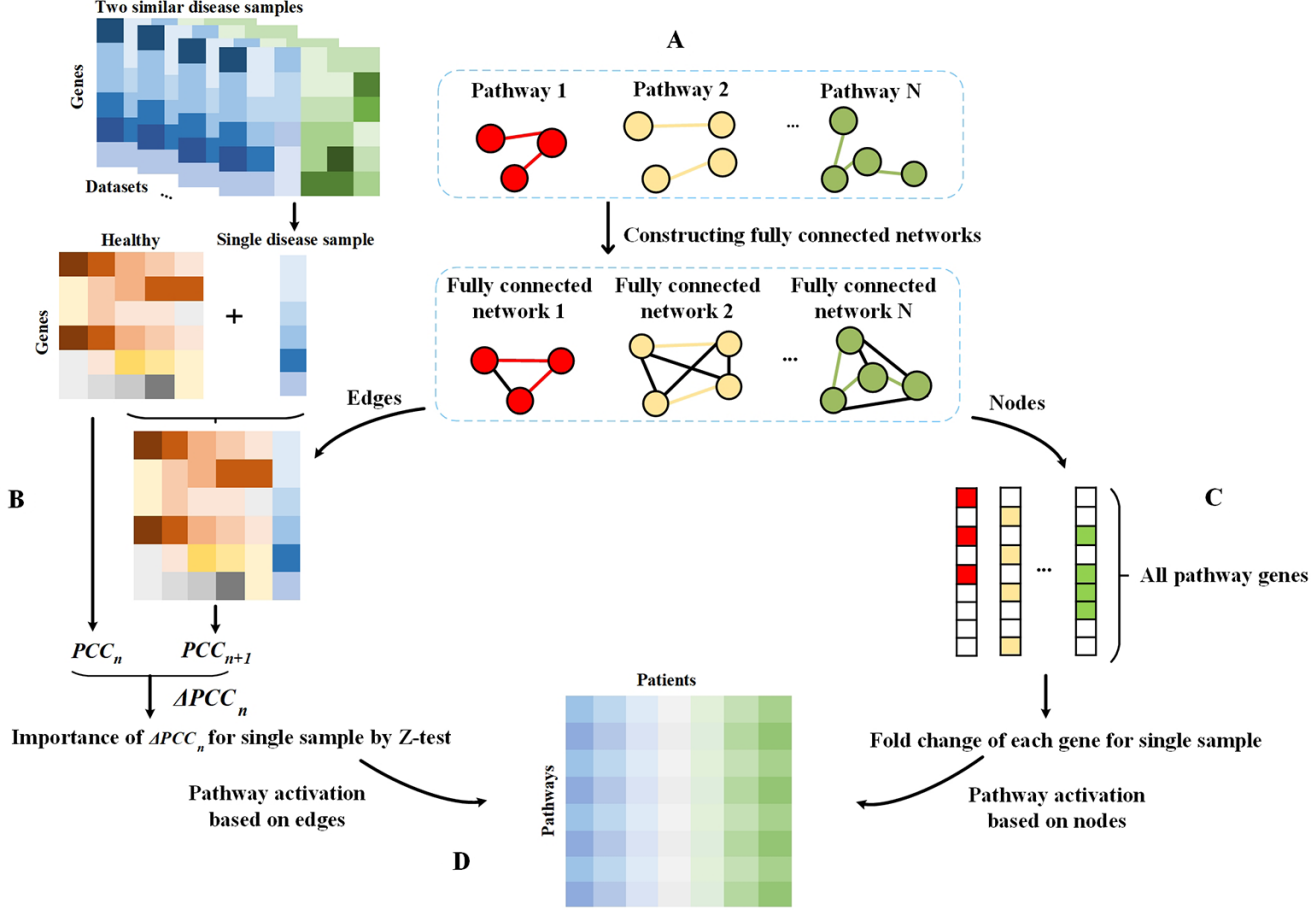
Schematic diagram of the framework. **(A)** Fully connected networks are derived from pathways. The colored edges represent the real interactions in pathways and are regarded as attention sets, and the black edges are artificially added to construct fully connected networks and regarded as the background sets. **(B)** Single sample theory for the evaluation of differential value of each edge in fully connected networks. **(C)** Obtaining all genes in pathways. For a pathway, genes on this pathway are considered as the attention set and others are treated as the background set. The fold change value of each gene in each disease sample relative to the normal sample is evaluated for subsequent analysis. **(D)** PASS expression matrix. For each pathway, the AUCpath is used to evaluate the enrichment of edges in a pathway as an AUC according to the ranking of all edges in a fully connected network, whereby all edges were ranked according to their Z-scores. For each pathway, the AUCpath is used to assess the enrichment of genes in the pathway as an AUC based on the ranking of all pathway genes, whereby all genes were ranked according to the gene expression data.

In this study, we first constructed a fully connected network for each pathway. For each dataset, we analyzed the condition-specific state for each disease sample based on the pathway and thus assessed the PASS features. The schematic diagram of our framework is shown in **Figure 1**.

#### Statistical Difference of Edges Between Single Disease Sample and Normal Samples

For each fully connected network, we used a group of *n* healthy samples to calculate the Pearson correlation coefficient (PCC) of each pair of genes as background value of the corresponding edge, denoted as *PCC_n_*. *PCC_n_* is defined as follows:

(1) $$PCC_{n}\left(x_{1},x_{2}\right)=\frac{E\left(x_{1}x_{2}\right)-E\left(x_{1}\right)E\left(x_{2}\right)}{\sqrt{E\left(x_{1}^{2}\right)-E^{2}\left(x_{1}\right)}\sqrt{E\left(x_{2}^{2}\right)-E^{2}\left(x_{2}\right)}}$$

where *x*_1_ and *x*_2_ are the expression profiles of a pair of genes that correspond to an edge, and *E* represents the operator of mathematical expectation.

Next, a single disease sample was added to the set of the normal samples, and the new PCC was calculated and denoted as *PCC_n_*_+1_. After that, the difference between background and interference values for the edges in each fully connected network could be quantified, which is represented as Δ*PCC_n_* (equal to *PCC*_*n*+1_−*PCC*_*n*_). The difference was derived from the influence of the newly added disease sample, thus it can reflect the specific characteristics of this single sample. Statistically, Δ*PCC_n_* obeys the volcano distribution. Therefore, the significance of Δ*PCC_n_* can be estimated by the hypothesis test Z-test. Z-value is calculated as follows:

(2) $$Z=\frac{\Delta PCC_{n}}{\left(1-PCC_{n}^{2}\right)/\left(n-1\right)}$$

#### Statistical Difference of Gene Expressions Between Single Disease Sample and Normal Samples

The statistical difference of genes between single disease sample and normal samples in the expression level was calculated by fold change:

(3) $$FC\left(x_{i}\right)=\frac{b}{\bar{a}}$$

where *b* represents the expression value of gene *x_i_* in the individual disease sample and $\bar{a}$ is the mean of expression values of gene *x_i_* over the *n* healthy samples.

#### Pathway Activation for a Single Sample

Based on the single sample analysis, we used AUCpath to estimate the activation of a pathway, which can evaluate the enrichment of an attention set as an area under the receiving operating characteristic curve (AUC) according to the ranking of all objects in a fully connected network. There were two sets, called the attention set and the background set. The attention set contained the subset of objects we considered as important, while the background set contained all the possible objects except important objects. We described the states of pathways from the aspect of genes and regulatory links.

From the perspective of edges, the input was the Z-value of all edges in each fully connected network, and the output was the activation of each pathway. The scoring approach was divided into two steps. First, the edges that exist in the pathway were regarded as an attention set (i.e., positive label), and the artificially added edges (in the step of the construction of fully connected network) were considered as the background set (i.e., negative label). Then, all edges in each fully connected network were ranked in ascending order of their Z-values. Second, AUC was applied to evaluate whether edges in a pathway are enriched in the top ranking, and we thus regarded the AUC value as the quantitative indicator of pathway activation. It is defined as follows:

(4) $$AUCpath=\frac{\sum_{i\in importantSubset}rank_{i}-\frac{m\left(1+m\right)}{2}}{m\times n}$$

where *rank_i_* represents the ranked position of the *i*-th edge of the attention set, *m* represents the number of edges in the attention set, and *n* is the number of edges in the background set.

Besides, considering that genes were also critical for mining effective information, we calculated the pathway activation from the perspective of genes. We first obtained all genes in pathways. For each pathway, genes on it were regarded as an attention set, and other genes were considered as the background set. Then, we assessed the enrichment of genes in the attention set as AUC based on the ranking of all genes, whereby all genes were ranked in ascending order according to their fold change between a single disease sample and normal samples.

After the evaluation of pathway activation from both nodes and edges, we obtained a matrix with PASS scores for pathways and patients.

## Results and Discussion

### Stronger Effectiveness of PASS Compared to the Representative Feature Engineering Methods

We built a comprehensive scheme to demonstrate the performance of our approach for distinguishing two similar diseases as well as compare them with other state-of-the-art feature engineering methods. We selected seven representative methods from three aspects: network-based, GO-based and pathway-based methods, that is, NetRank (Winter et al., 2012), stSVM (Cun and Fröhlich, 2013), comparative network stratification (CNS) (Zhang et al., 2017), principal component analysis (PCA) (Young and Craft, 2016), normal tissue centroid (NTC) (Young and Craft, 2016), gene expression deviation (GED) (Young and Craft, 2016), and probabilistic pathway score (PROPS) (Han et al., 2017). For a better comparison, we downloaded the PPI network from STRING database (http://string-db.org/) for NetRank, stSVM and CNS, and collected biological processes (BP) terms of Gene Ontology (GO) (http://www.geneontology.org/) for CNS.

NetRank (Winter et al., 2012) is a modification of PageRank. For a given gene, NetRank identifies the rank of a gene according to the rank of its neighbors in a PPI network. stSVM (Cun and Fröhlich, 2013) is a feature selection method which smooths the marginal statistic for differential expression genes by random walk kernel.

CNS (Zhang et al., 2017) is a framework that captures functional features for discriminating the disease states. Genes that are enriched by the same function (GO term) are aggregated through a flux balance model, and functional modules that maximize the distinction between UC and CD are then obtained.

For genes on each pathway, PCA (Young and Craft, 2016) compresses gene expression data and extracts principal components for the classification of disease status. For the hyperspace formed by genes on a particular pathway, NTC (Young and Craft, 2016) treats each disease sample as a point in the hyperspace and computes the Euclidean distance between the coordinates of disease samples and healthy samples. GED (Young and Craft, 2016) firstly uses the Kolmogorov–Smirnov test to capture genes that have the different distribution in normal and disease samples, and scores of those genes are then calculated based on the expression deviation in normal and disease samples. According to the scores, GED gives two features to each pathway, one for over-expression and one for under-expression. PROPS (Han et al., 2017) regards each pathway as a Gaussian Bayesian model. For each gene, after calculating the parameters in the model through normal samples, probabilistic pathway scores can be obtained using the loglikelihood values.

### Improved Discrimination of PASS Evaluated by Classification Performance Analysis

**Figure 2:**
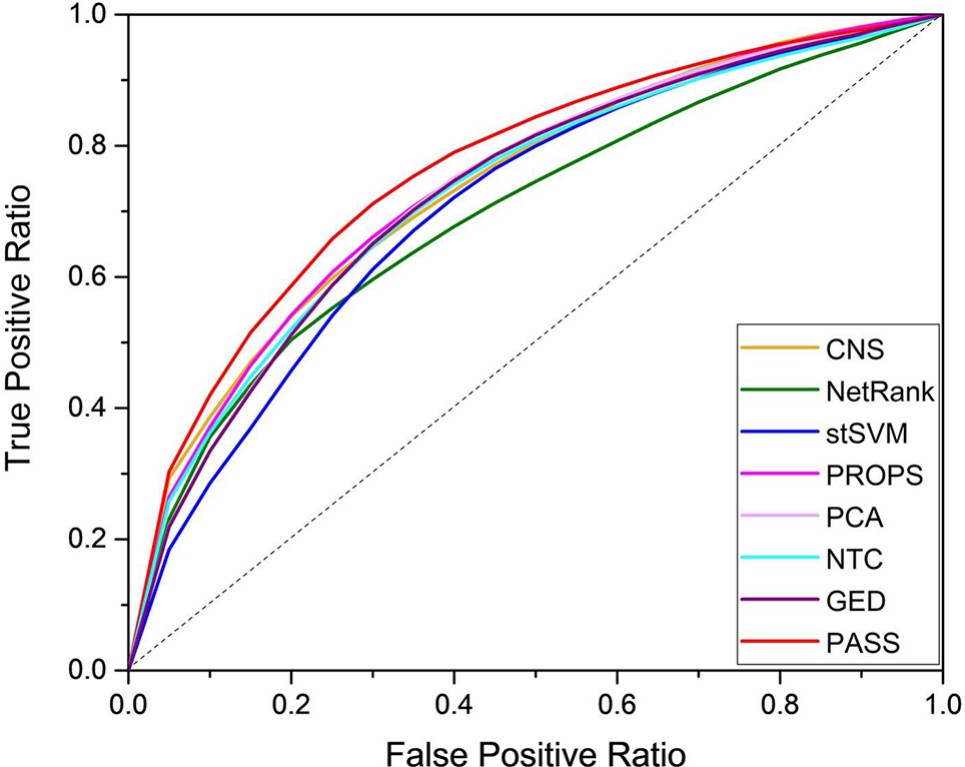
Aggregate ROC curves.

We used the random forest classifier to verify the classification results and applied three-fold cross-validation considering the small sample size of several datasets. For unbiased evaluation, we repeated these experiments for a total of 500 times for the entire datasets. The results of eight methods are shown as ROC curves and AUC corresponding to the ROC in **Figure 2** and **Table 2**, respectively. Although the AUC of PROPS on GSE3365 somewhat exceeded PASS, and the AUC of PCA on GSE16879 was equal to PASS, our method was more stable and more prominent than the other seven methods on the five datasets.

**Table 2:** Classification performance comparison on independent datasets.

Methods	GSE9686	GSE3365	GSE36807	GSE71730	GSE16879
PASS	0.94	0.77	0.78	0.74	0.72
NetRank	0.88	0.75	0.65	0.69	0.56
stSVM	0.88	0.72	0.75	0.71	0.55
CNS	0.91	0.75	0.75	0.70	0.69
PCA	0.91	0.66	0.73	0.69	0.72
NTC	0.89	0.72	0.75	0.67	0.68
GED	0.88	0.70	0.73	0.67	0.70
PROPS	0.88	0.78	0.70	0.73	0.67

### Analysis of Differential Pathways With Significance According to PASS

In order to validate the effectiveness of PASS features, we analyzed the differential pathways according to the PASS index. The p-value was calculated using two-sample t-test for the five datasets. **Supplementary Figure S1** shows the quantitative distribution of p-value of differential pathways based on the PASS scores for the five datasets. The pathway activation we defined can acquire lots of differential features with significance in two similar diseases, which indicates that the PASS index can widen the gap between UC and CD.

**Figure 3:**
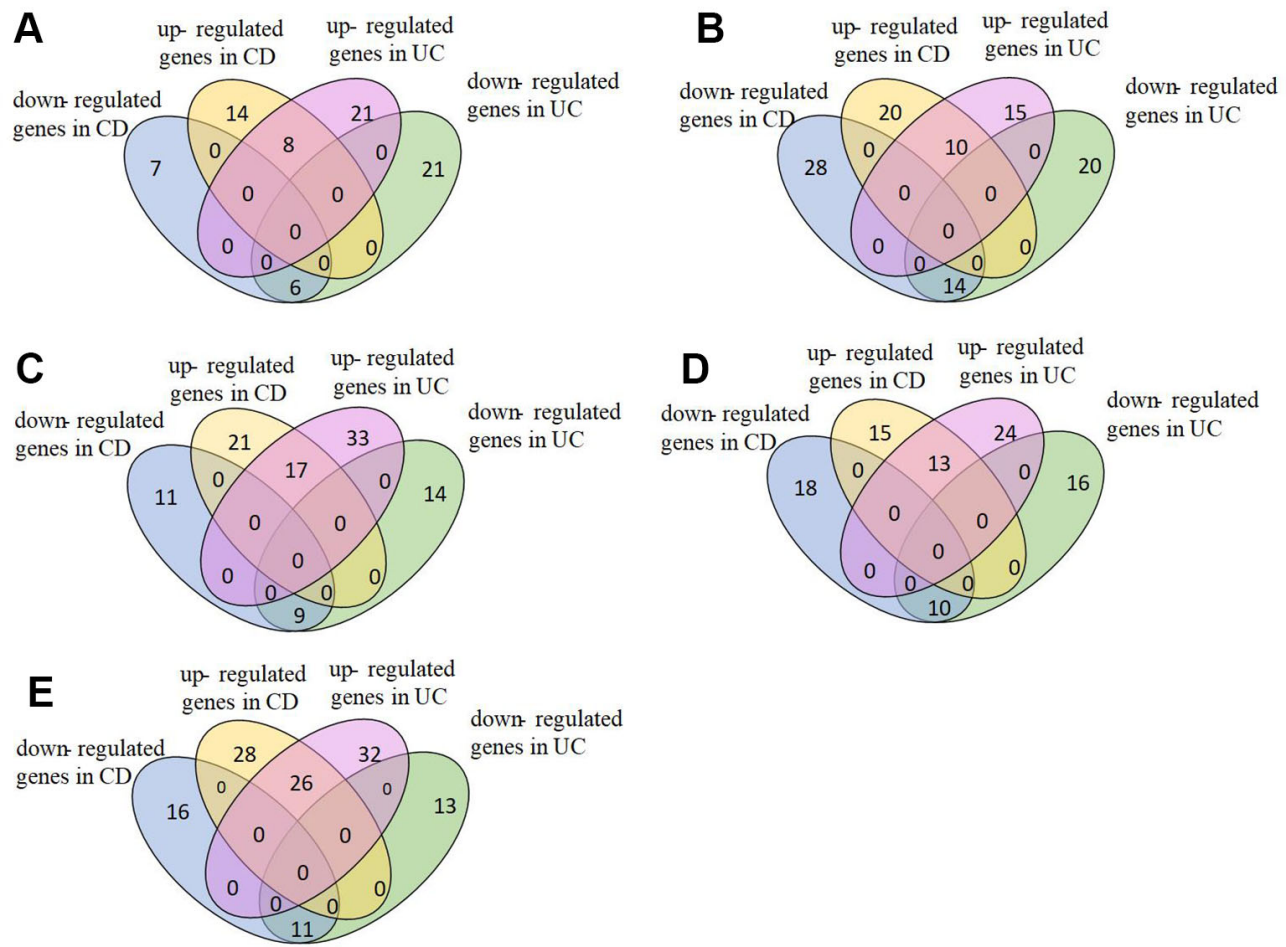
Expression of genes in Epstein-Barr virus infection pathway. **(A)**GSE9686,**(B)**GSE3365, **(C)**GSE36807, **(D)**GSE71730, **(E)**GSE16879.

We analyzed pathways that were differentially expressed (p-value < 0.05) on all the datasets (**Supplementary Table S1**). The majority of differential pathways have been shown to be related to IBD as reported in the literature (**Table 3**). These pathways not only demonstrate the metabolic and immune abnormalities of IBD, but they also reveal the pathogenesis of IBD from specific perspectives. Furthermore, the expression of genes in differential pathways related to IBD can reflect the changes in the course of disease. For the differential pathways associated with IBD, we analyzed the up-regulation and down-regulation of differentially expressed genes with significance in UC and normal samples, CD and normal samples. **Figure 3** shows the Venn diagrams of *Epstein-Barr virus infection* pathway, and others are shown in Supplementary **Figures S2**–**S10**. Most genes have the same regulatory relationship in UC and CD, but a small number of genes have different expressions. This also verifies that these two types of diseases are very similar, but there are differences between them.

**Table 3:** Differential pathways related to IBD.

Entry	Name	Reference
hsa05169	Epstein-Barr virus infection	(Yanai et al., 1999)
hsa00190	Oxidative phosphorylation	(Soderholm et al., 2000; Söderholm et al., 2002)
hsa00531	Glycosaminoglycan degradation	(Lee et al., 2008b)
hsa00730	Thiamine metabolism	(Mehanna et al., 2008)
hsa00860	Porphyrin and chlorophyll metabolism	(Jansson et al., 2009)
hsa04012	ErbB signaling pathway	(Ando et al., 2013)
hsa04340	Hedgehog signaling pathway	(Ghorpade et al., 2013)
hsa04920	Adipocytokine signaling pathway	(Karmiris et al., 2006)
hsa00062	Fatty acid elongation	(Belluzzi et al., 2000)
hsa00020	Citrate cycle (TCA cycle)	(Schicho et al., 2012)

**Figure 4:**
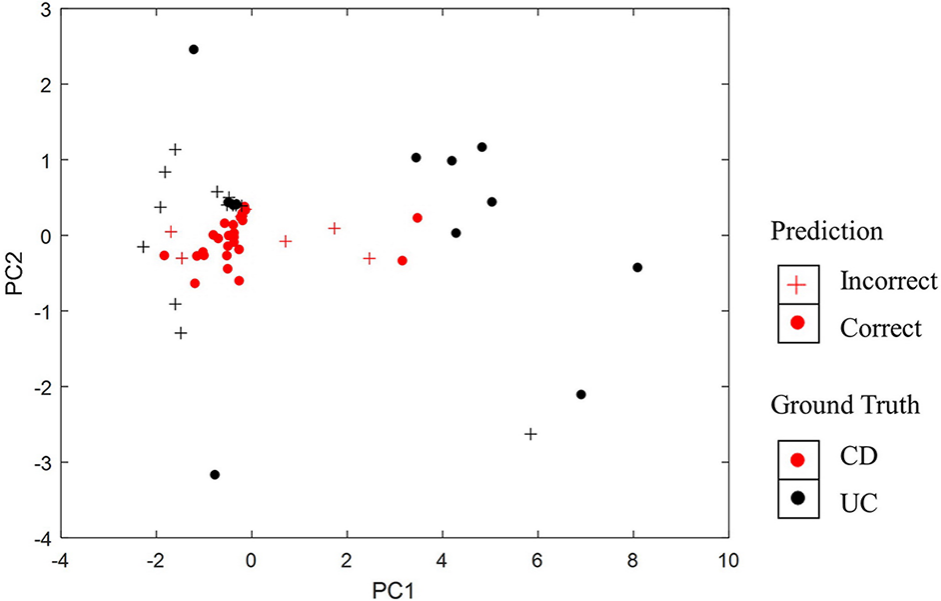
Visualization of classification results using the two principal components of PASS features.

Furthermore, we have visualized samples using the two principal components of our PASS features and overlaid the classification results from PASS model (**Figure 4**). The CD samples misclassified as UC and the UC samples misclassified as CD are mainly concentrated in the overlapping regions of the two types of diseases. However, some UC samples are more like CD samples, while some CD samples resemble UC samples, which leads to the misclassification of samples.

### Enrichment of Known Disease-Associated Genes

After choosing a p-value < 0.01 as the threshold of statistical significance, we obtained the significant differential pathways. Next, we analyzed the enrichment of the known disease-associated genes (DAGs) in differential expression pathways. DAGs relevant to UC and CD were collected from DisGeNET (Piñero et al., 2016), and a hypergeometric test was used to calculate the p-value of the enrichment of DAGs:

(5) $$P=1-\sum_{i=0}^{m-1}\frac{\left(\overset{M}{\underset{i}{}})(\overset{N-M}{\underset{n-i}{}}\right)}{\left(\overset{N}{\underset{n}{}}\right)}$$

where *N* is the number of genes in all pathways, *M* is the number of DAGs, *n* is the number of genes in the differential pathways, and *m* is the number of DAGs enriched in the differential pathways.

**Figure 5:**
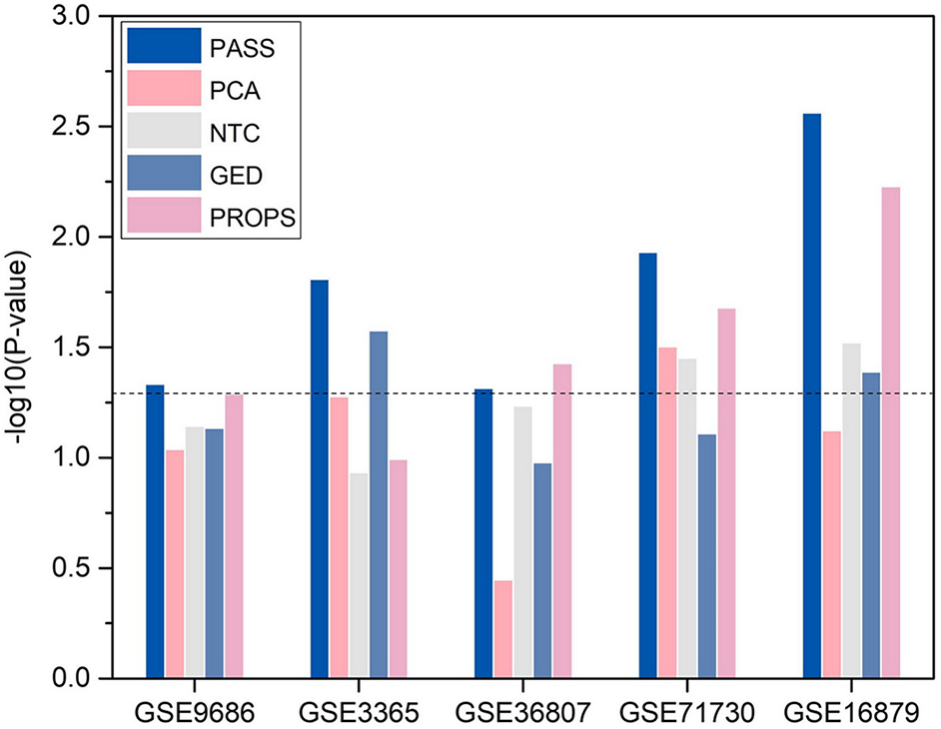
Enrichment of known disease-associated genes in the significant differential pathways.

For convenience, we transformed p-value to −*log*_10_(*p*−*value*). We compared the statistical significance of the enrichment of DAGs in the significant differential pathways identified by PASS index with other pathway-based indexes (**Figure 5**). It shows that, with the exception of being outperformed by PROPS in GSE36807, the differential pathways obtained from PASS values have the statistical significance of the enrichment of DAGs and have lower p-values than other methods in all datasets. This indicates that the PASS index has the ability to identify differential features enriched by DAGs.

## Conclusion

Complex diseases are not determined by a single gene, but by the combination of multiple genes, multiple factors, genetics, and the environment, similar complex diseases are more difficult to diagnose due to similar symptoms. In this study, we have presented PASS as a novel framework for classifying two main types of IBD from a single disease sample rather than a population of patients. For each pathway, we evaluated the difference between each patient and healthy sample from the perspective of genes and their interactions and calculated the pathway activation of individual samples. From the edge aspect, we constructed a fully connected network for each pathway, where edges in the pathway were regarded as the attention sets and artificially added edges were used as the background sets. Subsequently, we calculated the extent of perturbation of each edge based on single sample theory. From the node perspective, we collected all genes on all pathways. For each pathway, nodes on it were the attention set and others were the background set. Then, we evaluated the statistic difference of each node between single patient and healthy samples. Hereafter, we evaluated the pathway activation of each patient by computing the enrichment of attention set as an AUC according to the ranking of all genes or edges in the fully connected network.

We applied our method to UC and CD, which are two similar complex diseases of IBD. We compared PASS with seven state-of-the-art approaches (NetRank, stSVM, CNS, PCA, NTC, GED, and PROPS) on five IBD datasets. The results show that our PASS had the more discriminative power and was more stable than other seven methods. Besides, the PASS index can capture more differential expressed pathways with biological interpretability, which indicates that our PASS feature can widen the gap between UC and CD and aid researchers in comprehending the pathogenesis of these two similar complex diseases.

Our method can be applied to the classification of two similar diseases and has improved classification accuracy compared to seven state-of-the-art methods. However, due to the complexity and difficulty of similar complex diseases, there is still a space for improvement in the discriminative power. The performance of the PASS method relies on the all human pathway data and the topology of pathways, and more complete pathway information can better reveal the biological processes within cells and the statistic difference between a single disease sample and healthy samples calculated by our method can be also more accurate. With the rapid development of human interaction databases, we believe that the completer and more accurate pathway information could help to further improve the diagnosis of UC and CD.

## Data Availability Statement

Publicly available datasets were analyzed in this study. This data can be found at Gene Expression Omnibus (GSE9686, GSE3365, GSE36807, GSE71730, GSE16879).

## Author Contributions

XL and RZ conceived and designed the experiments. XL and XC performed the experiments and analyzed the data. XL wrote the paper. ML, JX, F-XW, and JW supervised the experiments and reviewed the manuscript.

## Funding

This work was supported in part by the National Natural Science Foundation of China (61832019, 61702054), the 111 Project (No. B18059), the Hunan Provincial Innovation Foundation For Postgraduate (CX20190123), and the Hunan Provincial Natural Science Foundation of China (Grant No. 2018JJ3568).

## Conflict of Interest

The authors declare that the research was conducted in the absence of any commercial or financial relationships that could be construed as a potential conflict of interest.
