# Supplementary material for: Evaluation of Pathway Activation for a Single Sample Toward Inflammatory Bowel Disease Classification
Source: Front Genet. 2020 Feb 5;10:1401. doi: 10.3389/fgene.2019.01401 (PMC7013001; doi:10.3389/fgene.2019.01401)
Supplement: Supplementary file 1 [file DataSheet_1.docx]

Supplementary Material

**Table S1** differentially expressed pathways on all the datasets

| Entry | Name |
| --- | --- |
| hsa00020  hsa00062  hsa00190  hsa00310  hsa00531  hsa00670  hsa00730  hsa00860  hsa04012  hsa04340  hsa04390  hsa04540  hsa04657  hsa04920  hsa04931  hsa05012  hsa05140  hsa05164 | Citrate cycle (TCA cycle) |
|  | Fatty acid elongation |
|  | Oxidative phosphorylation |
|  | Lysine degradation |
|  | Glycosaminoglycan degradation |
|  | One carbon pool by folate |
|  | Thiamine metabolism |
|  | Porphyrin and chlorophyll metabolism |
|  | ErbB signaling pathway |
|  | Hedgehog signaling pathway |
|  | Hippo signaling pathway |
|  | Gap junction |
|  | IL-17 signaling pathway |
|  | Adipocytokine signaling pathway |
|  | Insulin resistance |
|  | Parkinson disease |
|  | Leishmaniasis |
|  | Influenza A |
| hsa05169 | Epstein-Barr virus infection |


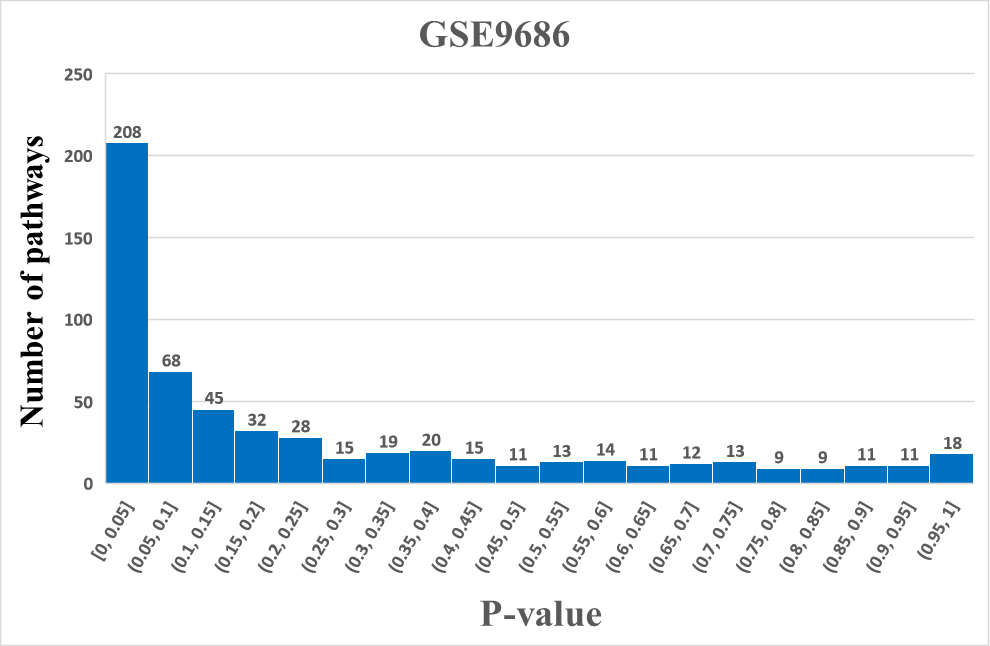

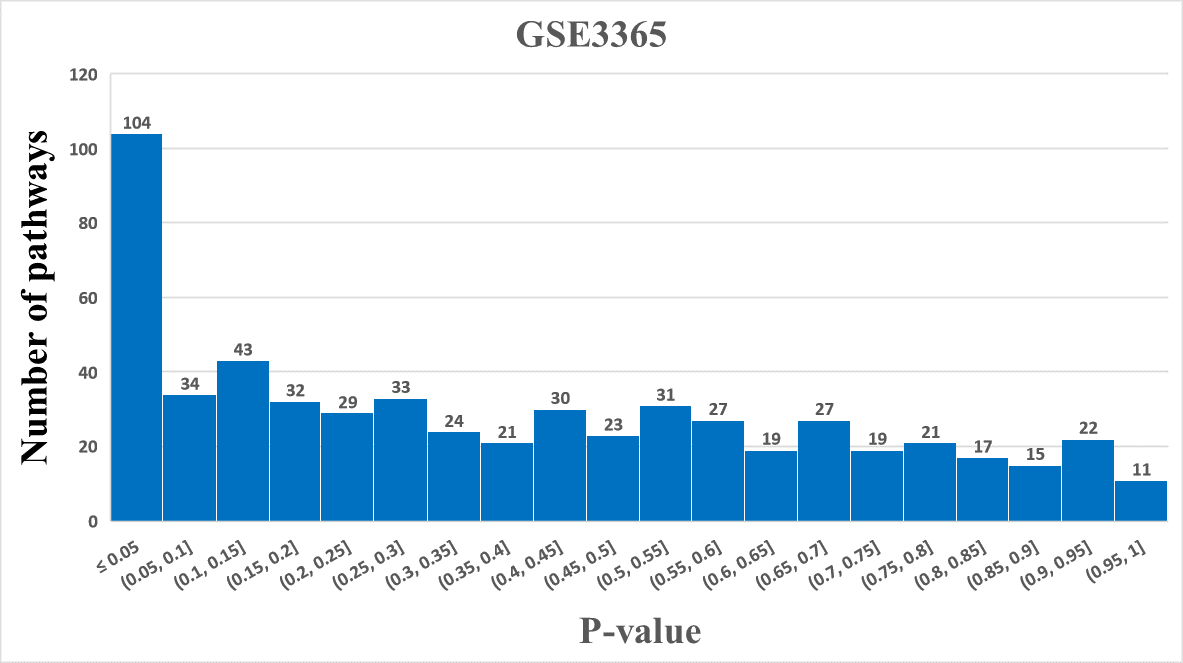


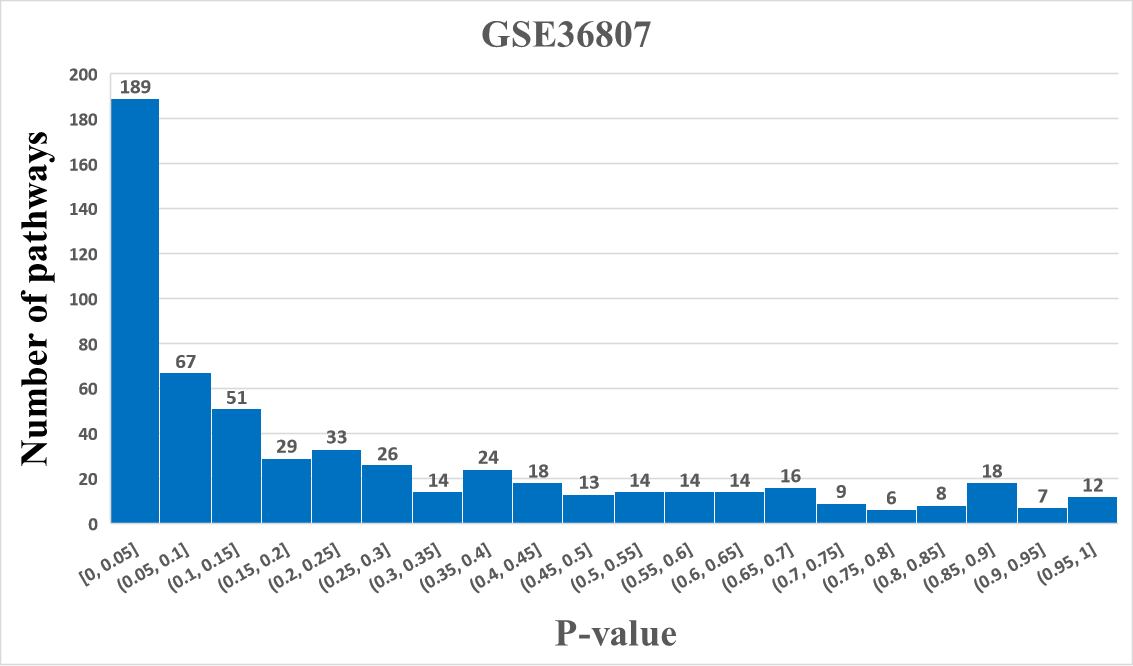

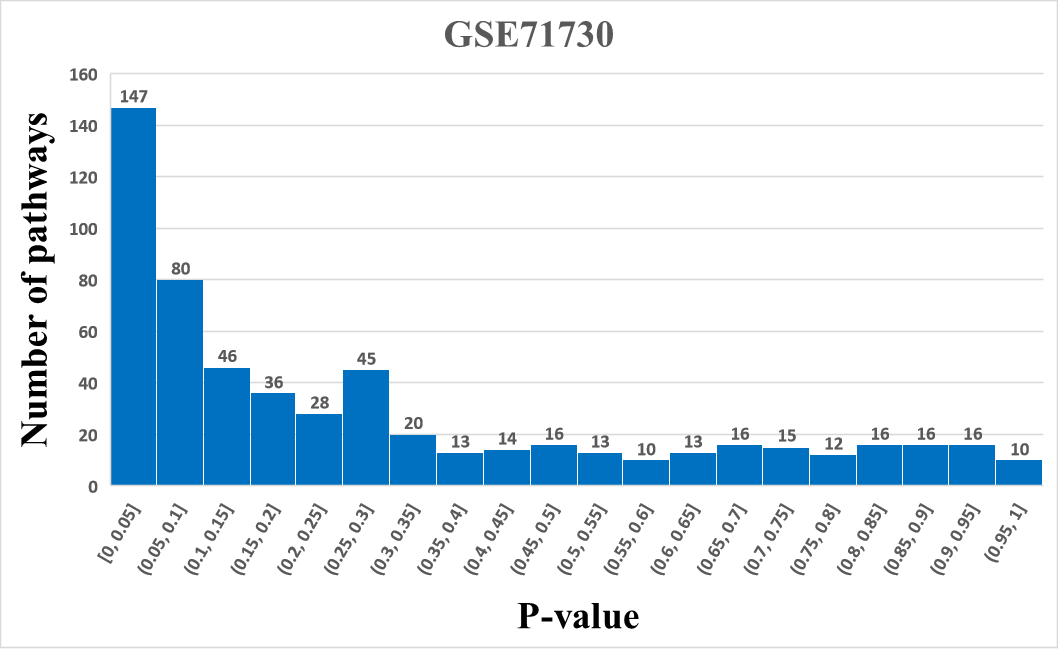


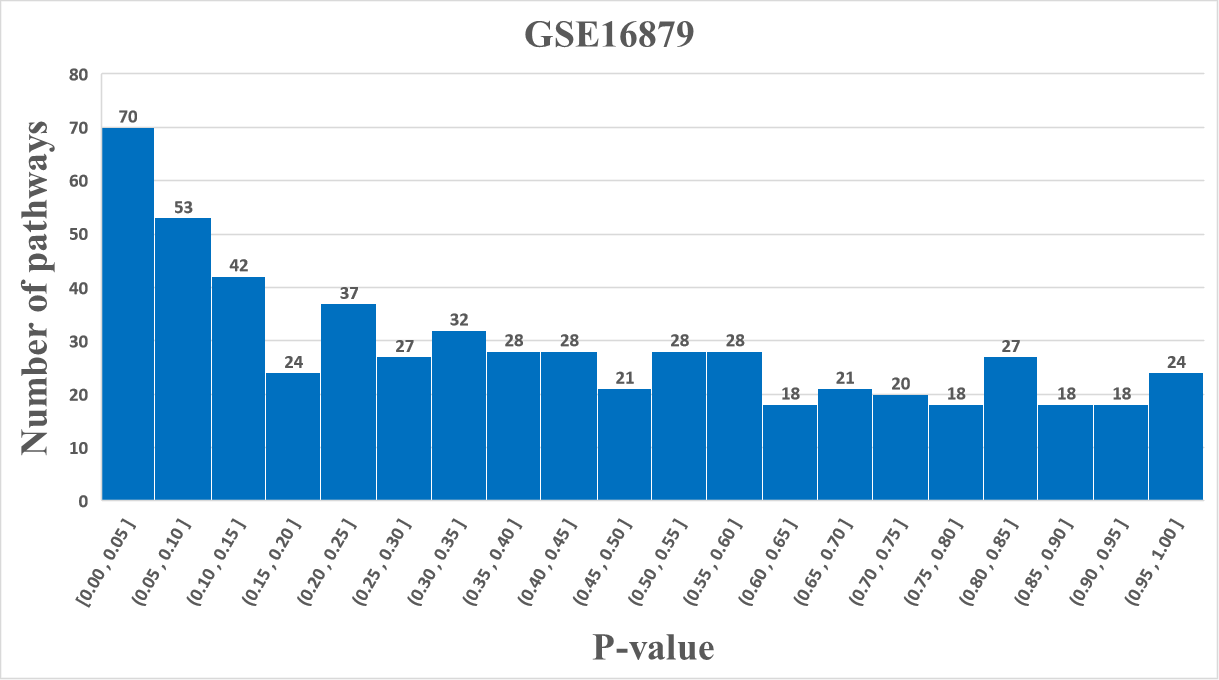


**Fig. S1** Quantitative distribution of p-value of differential expression pathways based on the PASS in the five datasets. The interval is set to 0.05, and the P-value is mostly less than 0.05 in the five datasets.


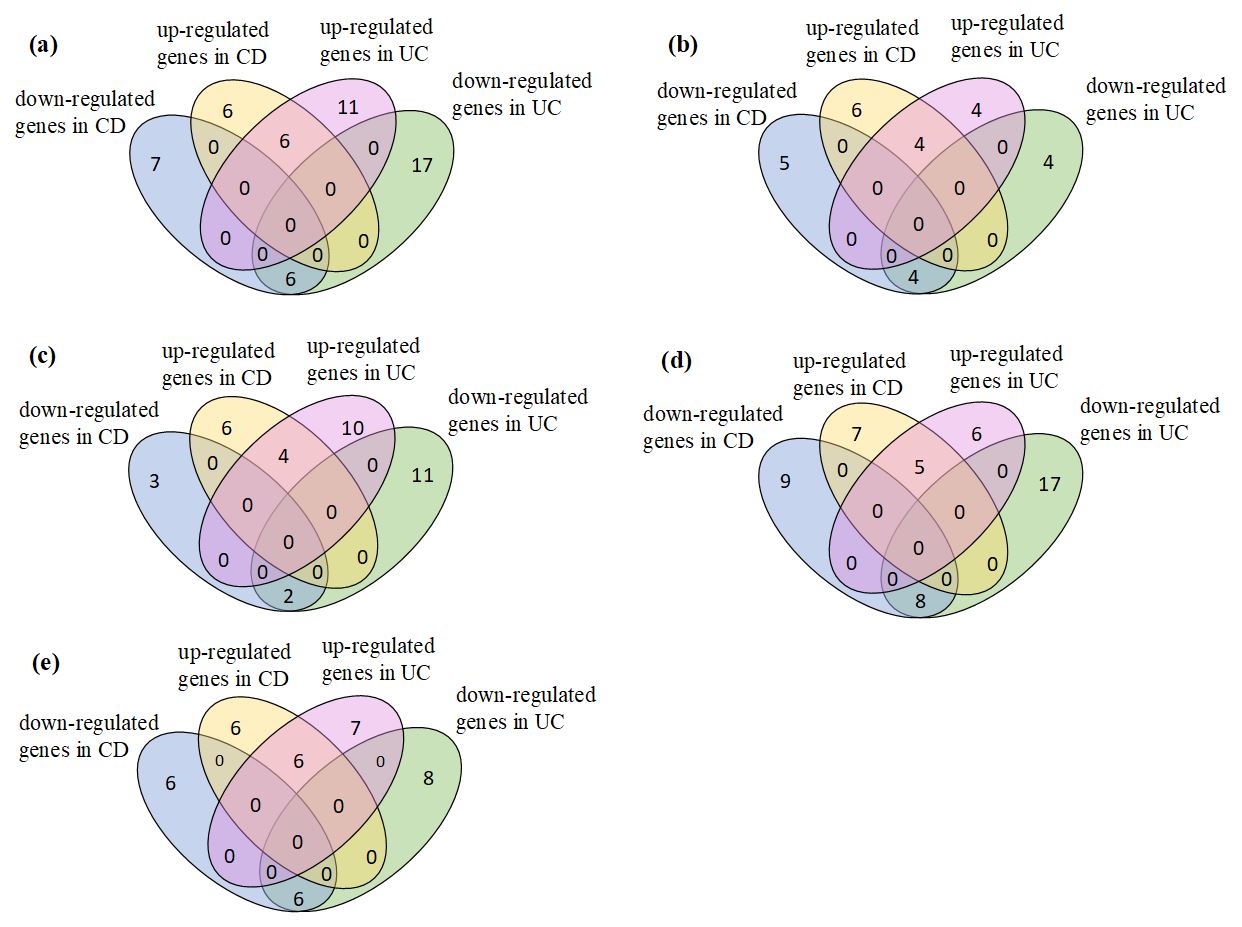


**Fig. S2** Expression of genes in Oxidative phosphorylation pathway. (a)GSE9686, (b)GSE3365, (c)GSE36807, (d)GSE71730, (e)GSE16879.


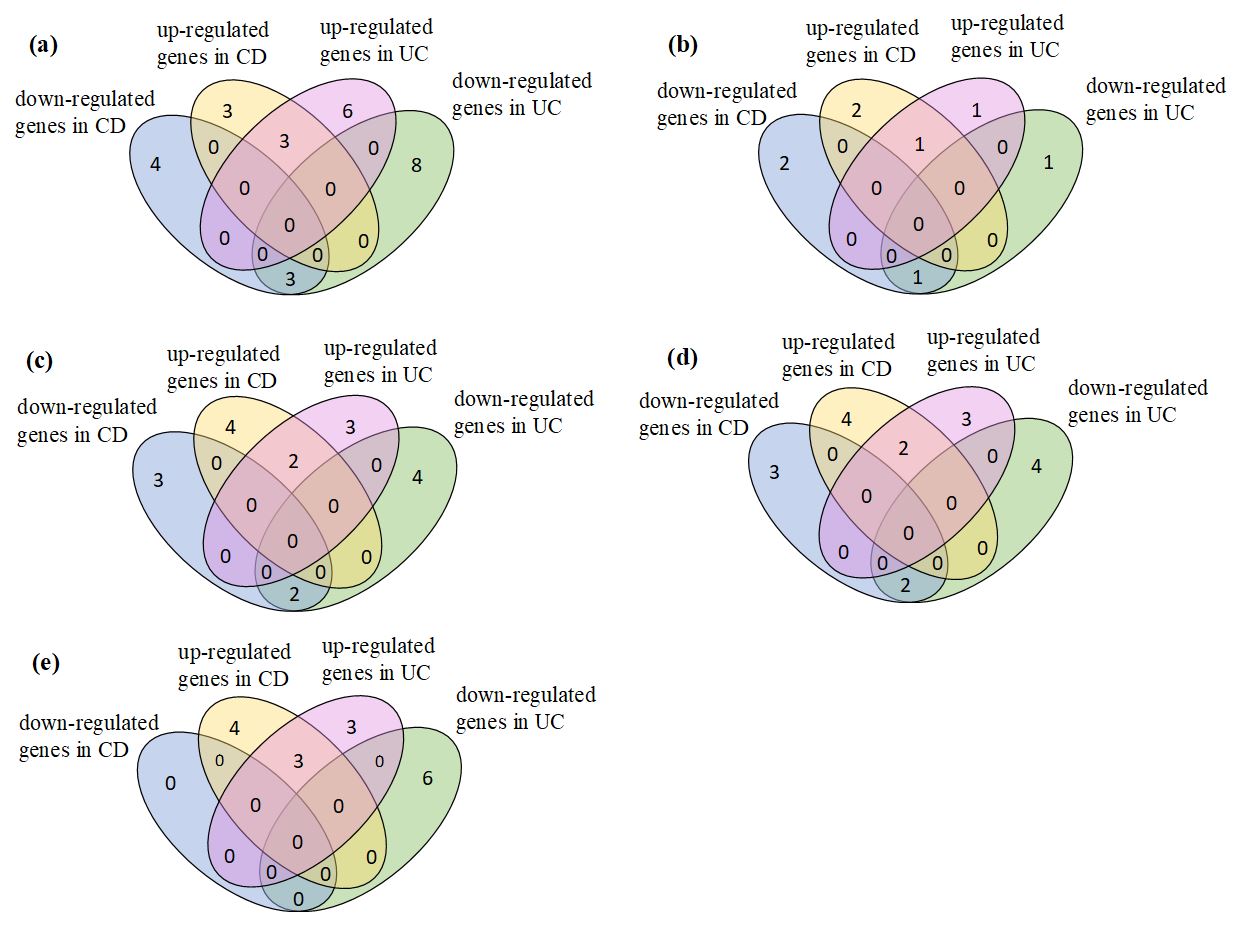


**Fig. S3** Expression of genes in Glycosaminoglycan degradation pathway. (a)GSE9686, (b)GSE3365, (c)GSE36807, (d)GSE71730, (e)GSE16879.


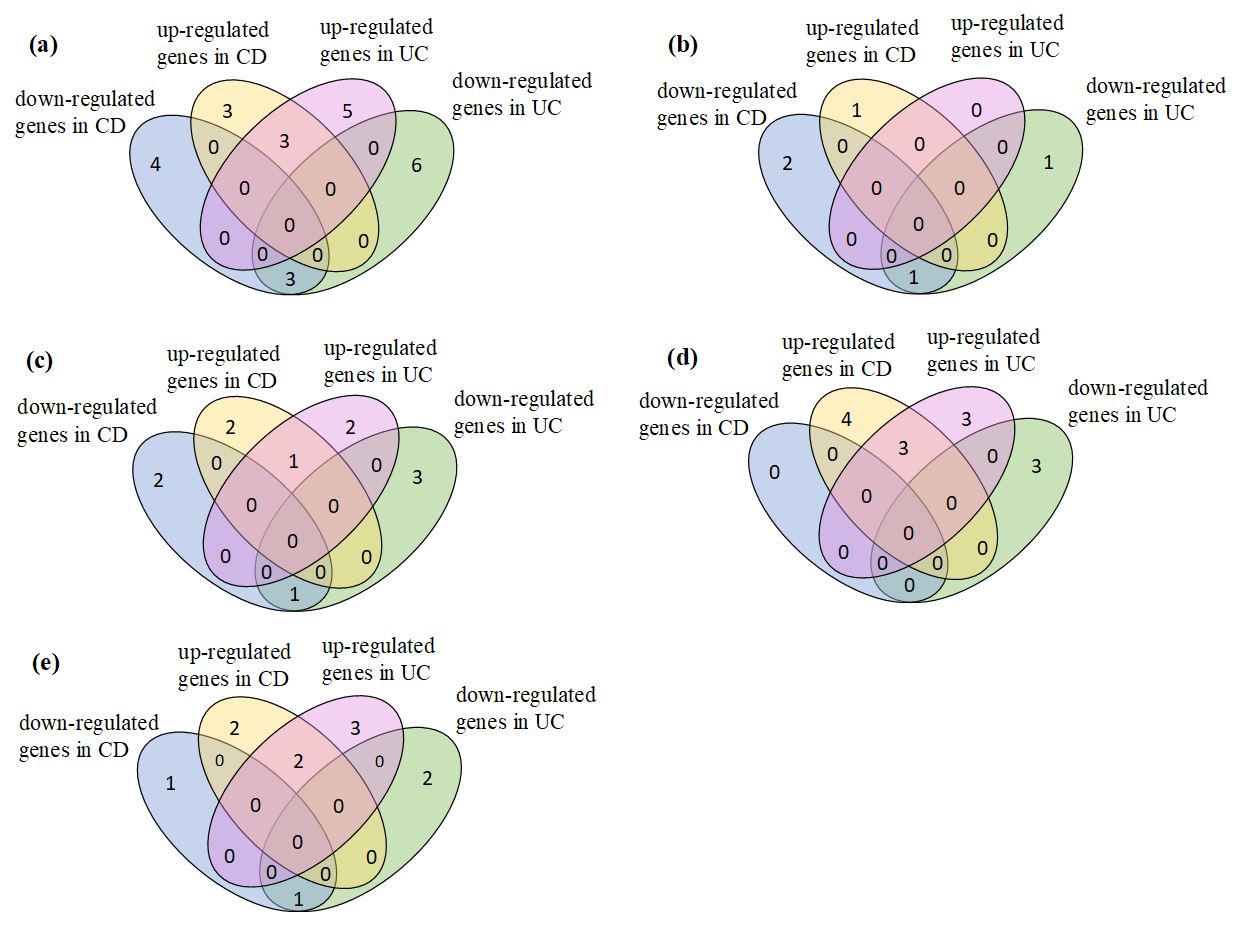


**Fig. S4** Expression of genes in Thiamine metabolism pathway. (a)GSE9686, (b)GSE3365, (c)GSE36807, (d)GSE71730, (e)GSE16879.


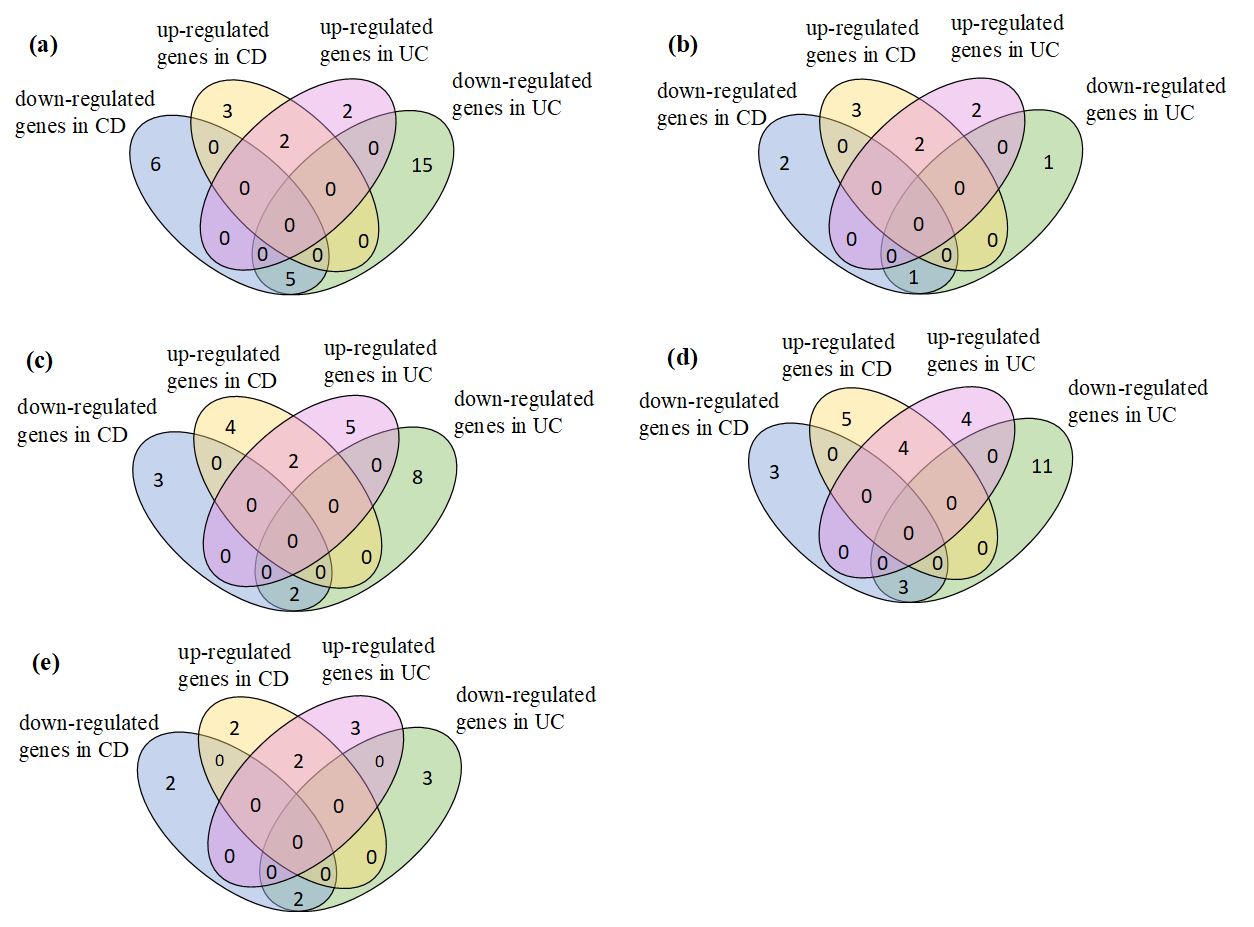


**Fig. S5** Expression of genes in Porphyrin and chlorophyll metabolism pathway. (a)GSE9686, (b)GSE3365, (c)GSE36807, (d)GSE71730, (e)GSE16879.


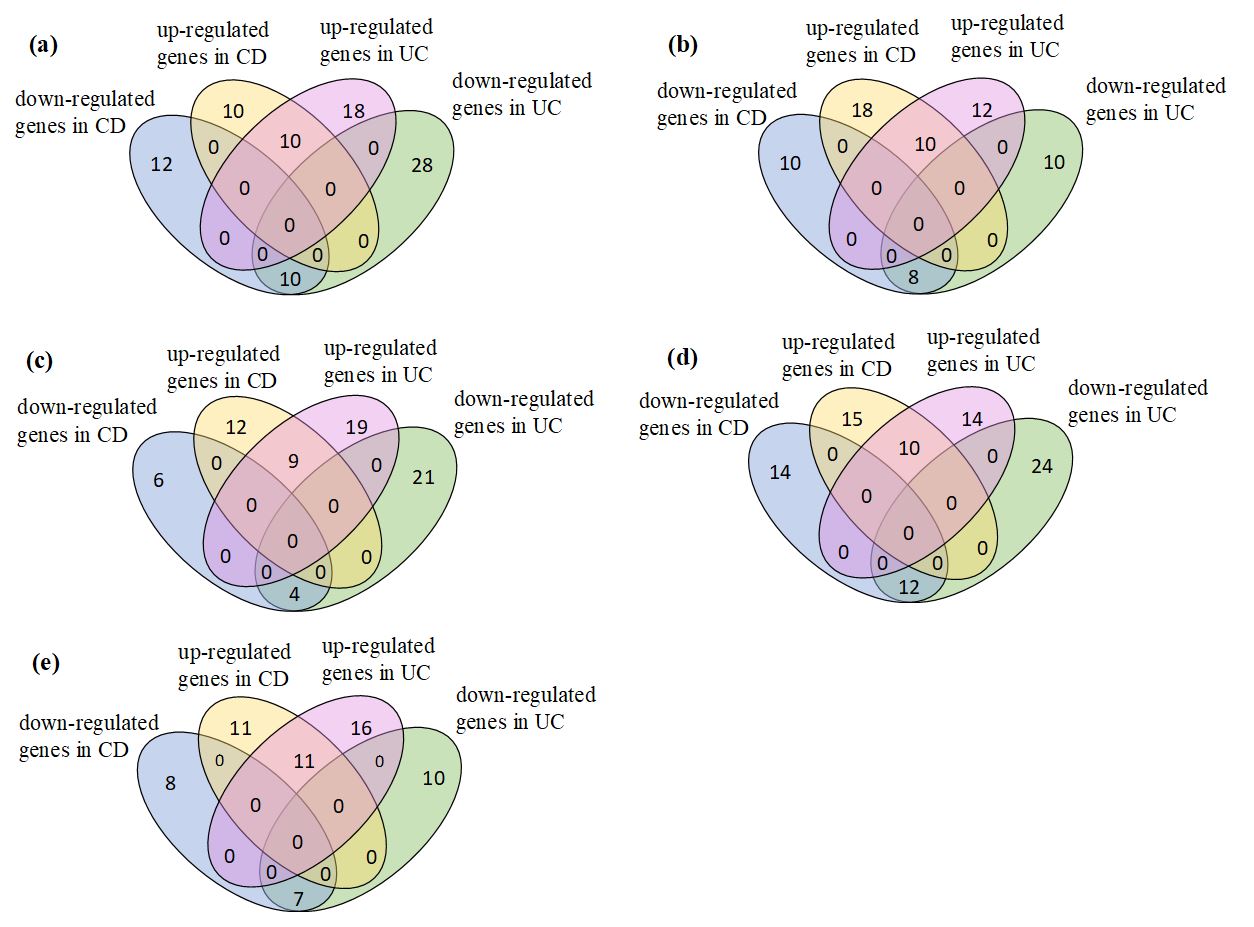


**Fig. S6** Expression of genes in ErbB signaling pathway. (a)GSE9686, (b)GSE3365, (c)GSE36807, (d)GSE71730, (e)GSE16879.


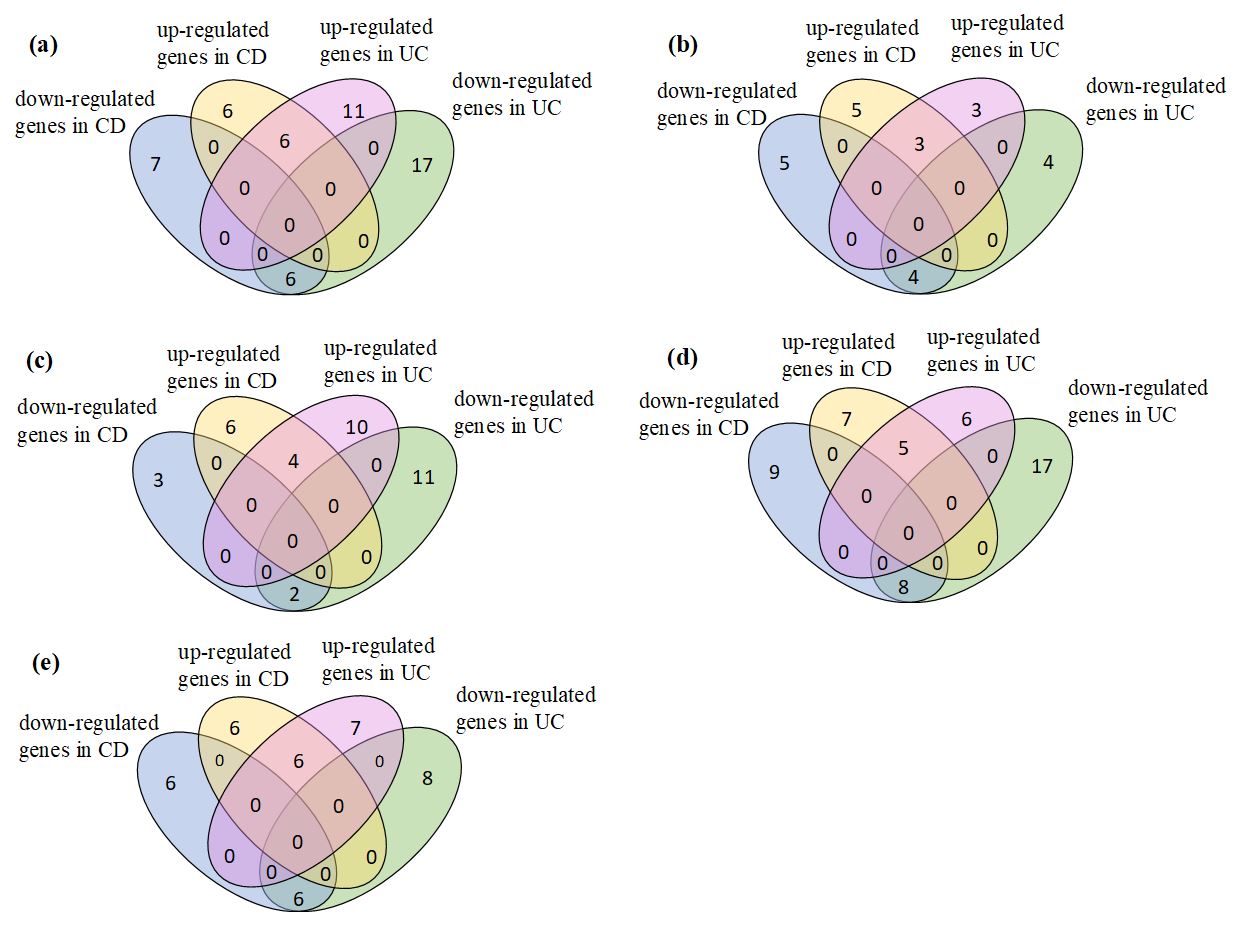


**Fig. S7** Expression of genes in Hedgehog signaling pathway. (a)GSE9686, (b)GSE3365, (c)GSE36807, (d)GSE71730, (e)GSE16879.


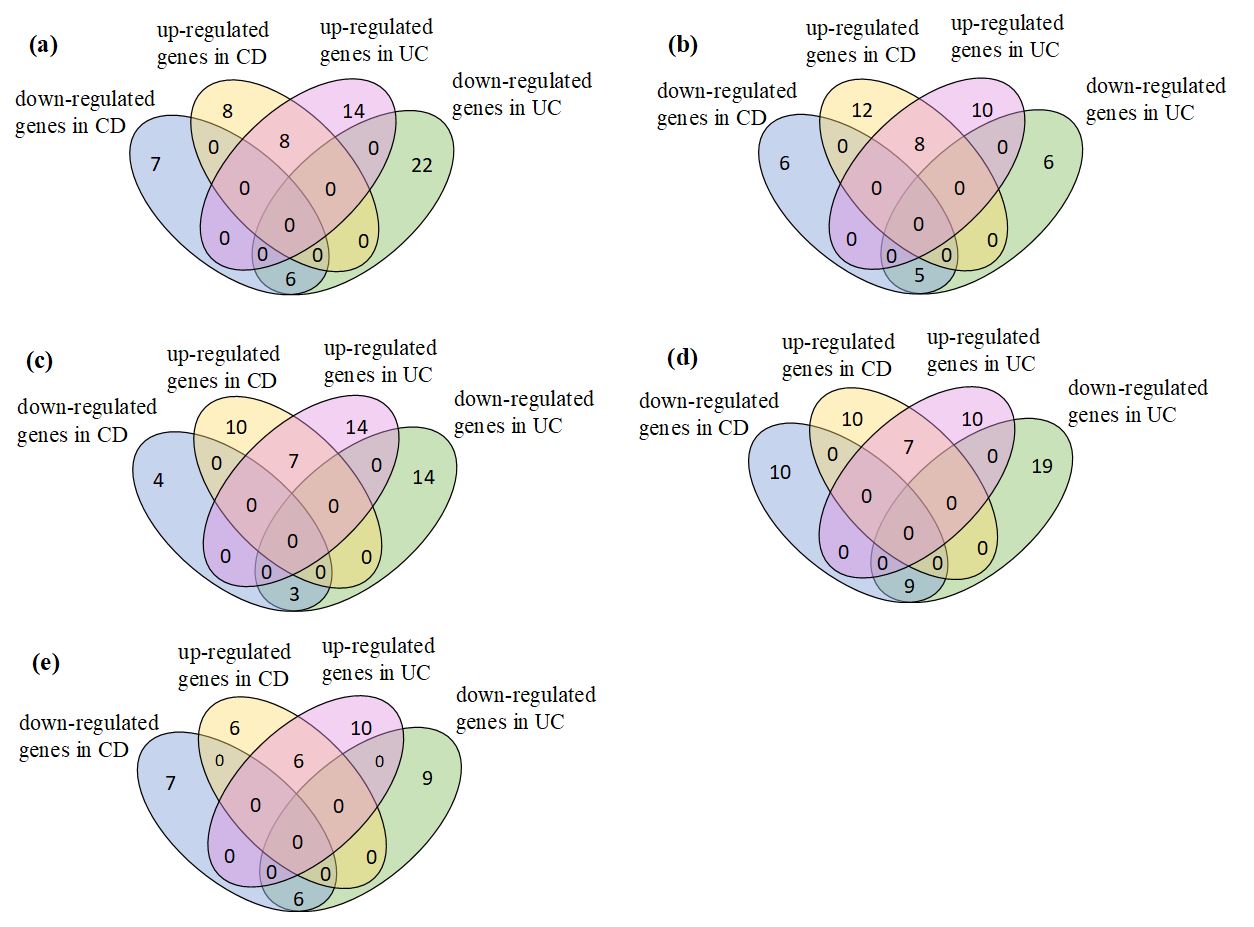


**Fig. S8** Expression of genes in Adipocytokine signaling pathway. (a)GSE9686, (b)GSE3365, (c)GSE36807, (d)GSE71730, (e)GSE16879.


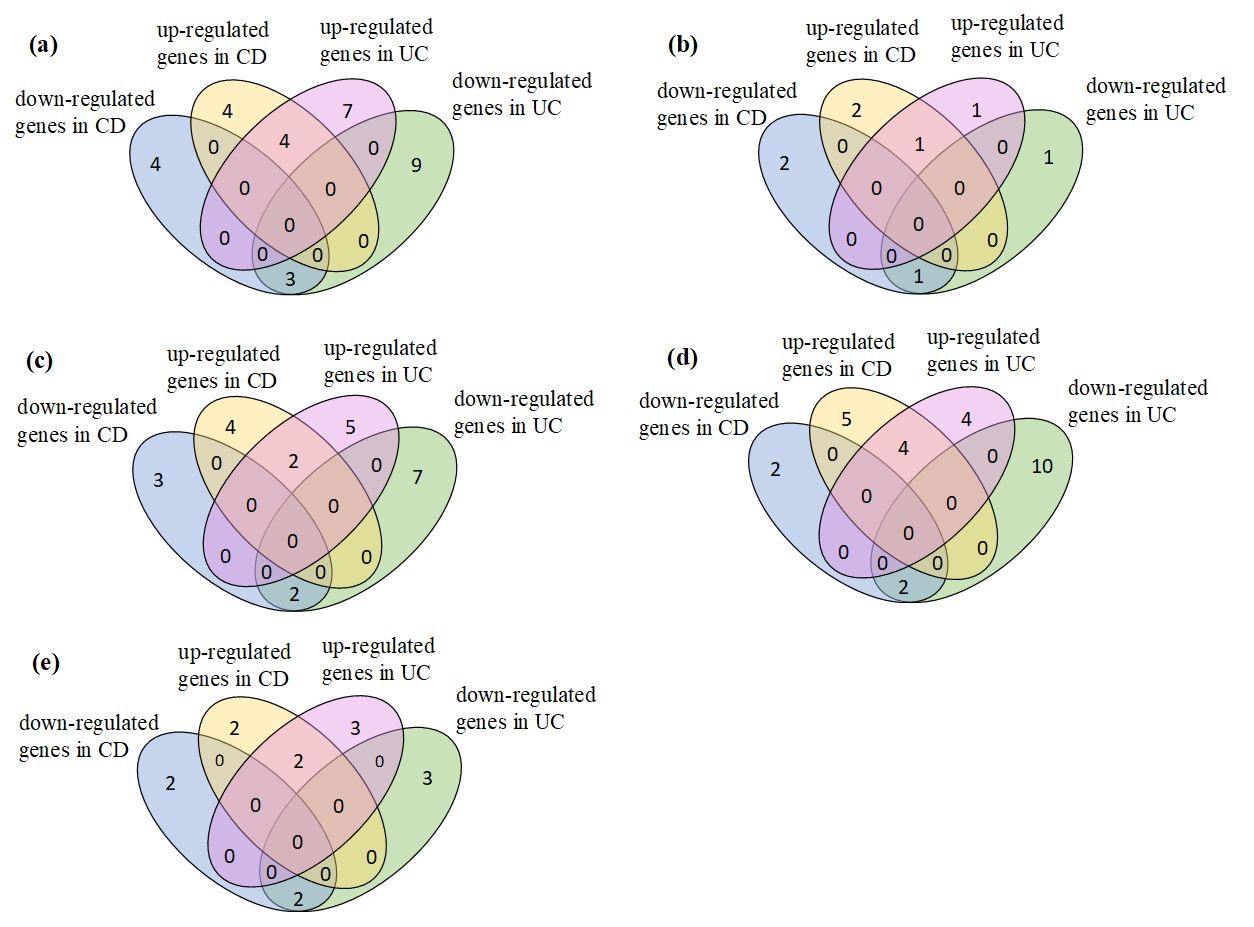


**Fig. S9** Expression of genes in Fatty acid elongation pathway. (a)GSE9686, (b)GSE3365, (c)GSE36807, (d)GSE71730, (e)GSE16879.


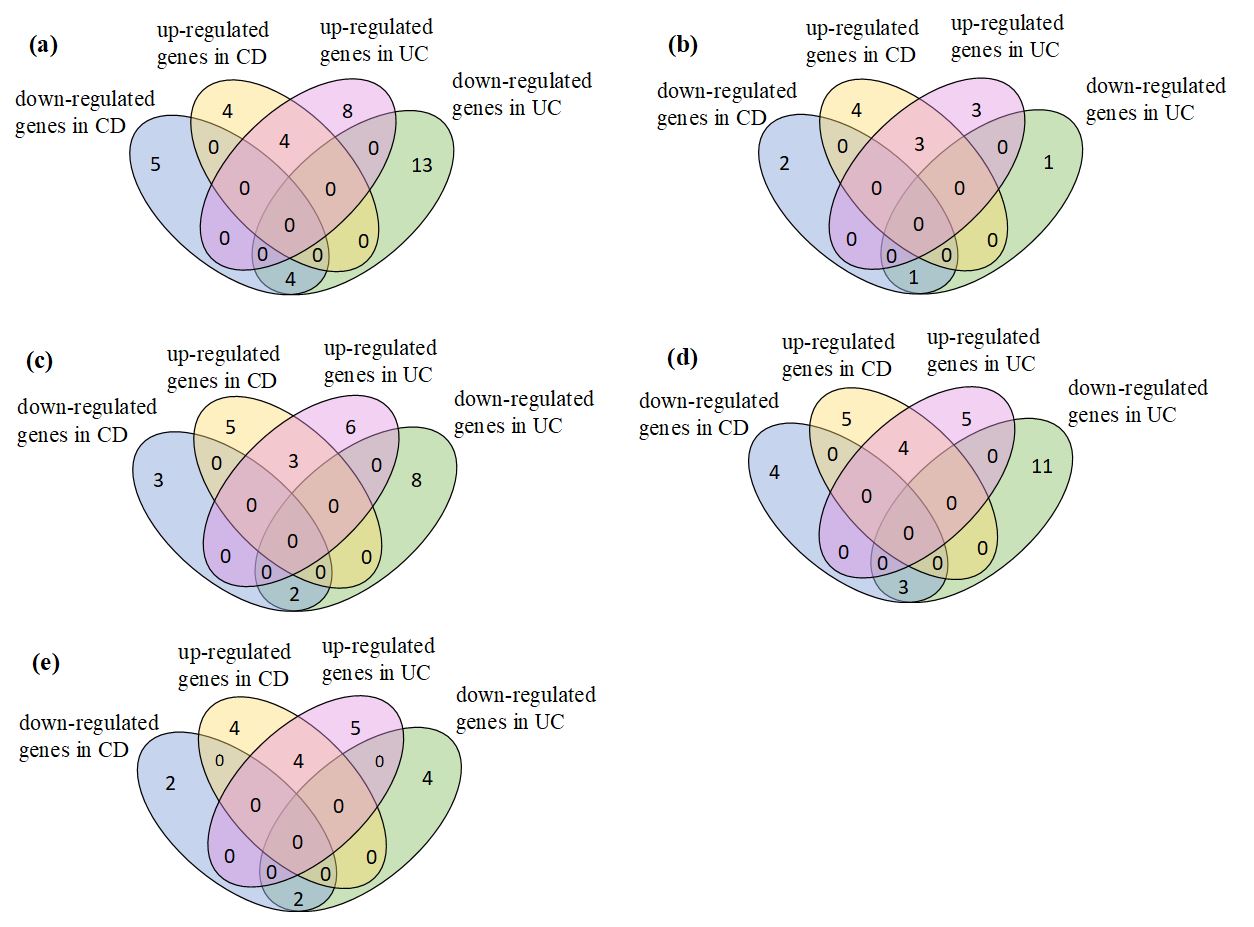


**Fig. S10** Expression of genes in Citrate cycle (TCA cycle) pathway. (a)GSE9686, (b)GSE3365, (c)GSE36807, (d)GSE71730, (e)GSE16879.
